# COVID-19 and the mental well-being of Australian medical students: impact, concerns and coping strategies used

**DOI:** 10.1177/1039856220947945

**Published:** 2020-08-10

**Authors:** Zaza Lyons, Helen Wilcox, Lianne Leung, Oliver Dearsley

**Affiliations:** Medical School, University of Western Australia, Australia; Medical School, University of Western Australia, Australia; Medical School, University of Western Australia, Australia; Medical School, University of Western Australia, Australia

**Keywords:** medical student, COVID-19, mental well-being

## Abstract

**Objective:**

Medical students are vulnerable to poor mental well-being. The recent COVID-19 pandemic has disrupted student life and had significant effects on curricula delivery at medical schools around Australia. The study aimed to assess the impact of COVID-19 on medical student mental well-being, assess concerns and determine activities used by students to help with the situation.

**Method:**

An online cross-sectional survey was designed. Questions focused on concerns and impact of COVID-19. The Kessler-10 (K10) measured psychological distress.

**Results:**

Two hundred and ninety-seven students participated with a 37.5% response rate. Mean K10 score was 20.6 indicating moderate psychological distress. There were no significant differences in K10 mean score or distress level (low, moderate, high, very high) between students in different years of the medical course. Deterioration in mental well-being since COVID-19 onset was reported by 68% students. Main negative impacts were on social connectedness, studies and stress levels. Concerns related to uncertainty about returning to normal and graduation. Common activities were using video chats, social media, exercise and hobbies.

**Conclusions:**

The impact of COVID-19 on mental well-being has led to legitimate concerns by students regarding their studies and progress through the medical course. We hope to minimise these disruptions, and reassure and support students to ensure that academic goals are achieved.

Medical school can be a stressful start to a professional medical career. Evidence shows that medical students are particularly vulnerable to poor mental well-being and have higher rates of mental illness and psychological distress compared to the general population.^1^ Meta-analysis shows that depressive symptoms among medical students are high, with a 27.2% prevalence reported by Rotenstein (2017).^2^

A number of factors contribute towards medical student stress. Commonly reported stressors identified from the literature include academic factors such as feeling unprepared for exams; the organisational structure of medical courses including timetabling, inadequate and delayed feedback of assessment results; and poorly organised placements.^3,4^ Psychological distress often persists throughout medical school and can have adverse consequences such as academic burnout, an increased risk of dropout and sleep problems.^4^

The recent COVID-19 global pandemic has affected students’ lives on many different levels and had significant effects on delivery of the teaching curriculum across the *University of Western Australia (UWA)* 4-year graduate Doctor of Medicine (MD) course. The speed at which the community lockdown and subsequent changes to the medical course occurred resulted in many students struggling to adjust to online learning and other curriculum changes that were implemented in response to government lockdown measures. The abrupt cancellation of previously timetabled face-to-face tutorials, clinical placements and patient contact further contributed to disruption of normal studies.

In addition to teaching-related changes, other impacts such as loss of peer interaction and social connectedness, and financial stressors including loss of part-time employment caused by the economic consequences of the pandemic have potential to impact negatively on student well-being and cause further psychological distress, disrupt daily life and medical studies. It is important to actively assess and monitor this to mitigate negative impacts and provide appropriate support to students. The study aimed to: (a) determine the impact of COVID-19 on mental well-being of UWA medical students; (b) determine specific concerns of students and (iii) determine activities and strategies used by students to help with the COVID-19 situation.

## Method

An online cross-sectional survey using the Qualtrics platform was designed. The survey was developed in collaboration with academic staff and student representatives. Survey questions included demographic information; self-rated assessment of current mental well-being; the Kessler 10 (K10), questions regarding the impact of COVID-19 on various areas of life, concerns about COVID-19 and activities and strategies used to help with the situation. The questionnaire is available on request.

All MD students were eligible to participate. The survey was promoted through the learning management system and social media. Participation was voluntary and completion of the survey implied consent. No identifying details were collected. To avoid multiple responses submitted from the same subject, the survey was configured so that it could only be taken once.

Statistical analysis was conducted using SPSS (version 26). Descriptive statistics analysed demographic characteristics, and assessed the impact of COVID-19 on students’ life and concerns about the COVID-19 situation. K10 data were analysed descriptively. A one-way ANOVA tested significant differences between mean K10 score and MD year group. K10 scores were grouped into low, moderate, high and very high distress categories. Differences in levels of psychological distress between MD years was tested using the Kruskal–Wallis H test.

The study was approved by the UWA Human Research Ethics Committee.

## Results

### Demographics

Across the four MD years, 297 students participated with a 37.5% response rate. Overall, 46% of first year students (MD1) responded, 39% of second year students (MD2), 36% of third year students (MD3) and 34% of final year students (MD4). The mean age of respondents was 24 years, range 20–46, median 23 years. In terms of location prior to commencement of medical studies, 61% were from the Perth metropolitan area, 24% from rural/regional areas, 8.5% were international students and 6.5% from interstate.

### Psychological distress and mental well-being

To analyse K10 data, Australian Bureau of Statistics cut-offs were used, where a score of 10–15 indicates low psychological distress; 16–21, moderate distress; 22–29, high distress; and 30–50, very high distress.

Across all years, the mean K10 score was 20.6, indicating a moderate level of psychological distress. MD1 students had the highest mean score, 23.1, and MD3 students had the lowest, 19.8. The mean score for MD2 was 20.1 and for MD4 students 20.6. A one-way ANOVA found no significant differences in mean K10 scores between year groups, *F*(3, 290) = 2.394, *p* = 0.69. An independent t-test found that the mean K10 score was significantly higher for female students (21.3) compared with males (19.4), t(291) = −2.47, *p* = 0.014.

K10 data were categorised into low, moderate, high and very high psychological distress. The majority of students across all year groups were in the ‘moderate’ category (37%). However, 11% of students scored in the ‘very high’ category. MD1 had the highest proportion of students in the ‘very high’ category, 24% compared with 10% of MD4 and 9% for both MD2 and MD3. A Kruskal–Wallis ANOVA indicated that there were no statistically significant differences between the levels of distress in students in MD1 (Mean rank = 165.82), MD2 (Mean rank = 142.39), MD3 (Mean rank = 141.42) and MD4 (Mean rank = 150.24), H (corrected for ties) = 3.882, df = 3, *N* = 294, *p* = .275, Cohen’s *f* = 0.12. Table 1 shows K10 results.

**Table 1.** Kessler 10 scores across all year groups

MD year	Mean (SD)	Low distress (10–15) *n* (%)	Moderate distress (16–21) *n* (%)	High distress (22–29) *n* (%)	Very high distress (30–50) *n* (%)
MD1	23.1 (9.1)	9 (23.7)	11 (28.9)	9 (23.7)	9 (23.7)
MD2	20.1 (6.5)	27 (31)	27 (31)	25 (29)	8 (9)
MD3	19.8 (6.0)	22 (27)	32 (39.5)	20 (24.5)	7 (9)
MD4	20.6 (5.6)	18 (20.5)	39 (44.5)	22 (25)	9 (10)
All years	20.6 (6.6)	77 (26)	110 (37)	77 (26)	33 (11)

In terms of self-reported change in mental well-being since COVID-19 onset, a worsening of well-being was reported by 68% of students, 26% reported no change and 6% improved.

### Impact of COVID-19 on different areas of life

**Figure 1.**
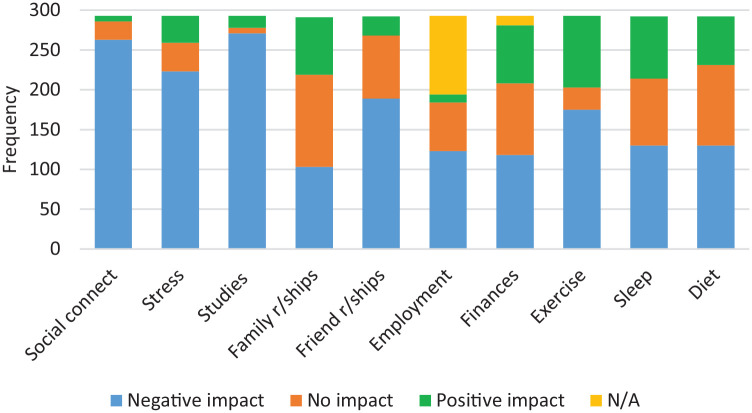
Impact of COVID-19 on different areas of life.

Respondents were asked to rate the impact of COVID-19 (negative impact, no impact, or positive impact) on different areas of life. As shown in Figure 1, the main negative impacts were on social connectedness, medical studies and stress levels. The main positive impacts were around family relationships, exercise and sleep.

### Concerns related to COVID-19

Respondents also reported their concerns regarding COVID-19. Across all years, the main concern was the impact on studies (81% of respondents), followed by uncertainty about a return to normal (72%). Other concerns were family member testing positive (62%) and being in self-isolation (42%). Table 2 shows each area of concern, the number and percentage of respondents in each year group who selected each choice. Concerns about graduation differed between years with only 10% of MD1 students expressing concern about this compared to 20.5% of MD2 students, 42% of MD3 and 67.5% of MD4 students. Concerns about the impact of COVID-19 on studies were highest among MD1 students (81%). MD3 students expressed more concern about uncertainty of when things would return to normal (80%) compared with students of other years.

**Table 2.** Concerns about COVID-19 across year groups

	Impact on studies *n* (%)	Getting back to normal *n* (%)	Family test positive *n* (%)	Being in self-isolation *n* (%)	Graduation concerns *n* (%)	Travel restrictions *n* (%)	Self-test positive *n* (%)
MD1	33 (87)	25 (66)	26 (68)	20 (53)	4 (10)	9 (24)	9 (24)
MD2	71 (81)	59 (67)	57 (65)	41 (45.5)	18 (20.5)	25 (23)	19 (22)
MD3	67 (83)	64 (80)	46 (57)	33 (41)	34 (42)	35 (43)	13 (16)
MD4	69 (77.5)	65 (73)	56 (63)	31 (35)	60 (67.5)	31 (35)	25 (28)
Total	240 (81)	213 (72)	185 (62)	125 (42)	116 (39)	100 (34)	66 (22)

Additional comments about concerns regarding the COVID-19 situation were provided by some students. These included issues around teaching and learning, for example worries about level of clinical skills and experience, and being unprepared for the workplace; concerns regarding their mental well-being; concerns specifically related to COVID-19 – for example, potential exposure while on placement or people in the community not adhering to social distancing regulations; and concerns about loss of social contact with family due to lockdowns.

Students were asked about activities and strategies used to help with the COVID-19 situation. Video chats and social media apps were the most commonly reported strategies – 87.5% and 62%, respectively. Mindfulness and meditation were used by 36%. Some additional comments were also provided. These included exercise and fitness, such as jogging, cycling, indoor exercising; taking up new hobbies and revisiting old hobbies; focusing on routine and goal setting; creative activities such as playing and listening to music, reading, watching movies; cooking and baking; playing video games; attending online church services and prayer. Table 3 shows these results.

**Table 3.** Activities and strategies used to help with mental well-being

	Number of respondents	% of respondents
Video chat	241	82%
Social media apps	162	55%
Mindfulness/meditation	97	33%
GP	27	9%
Student society activities	25	8.5%
Clinical mentor	23	8%
Psychologist	20	7%
Web/phone-based services	12	4%
Counsellor	10	3.5%
Psychiatrist	4	1.5%

## Discussion

Results of this study have shown that students are currently experiencing moderate levels of psychological distress, and two-thirds reported a deterioration in mental well-being since the onset of the COVID-19 pandemic in Australia (mid-March 2020). While psychological distress is common among medical students, particularly females, K10 results from this study show that more students were scoring in the ‘very high’ distress category compared with other studies, including the ‘beyondblue’ survey of doctors and medical students.^1,5^ Inevitably, an elevated level of stress has impacted negatively on their lives and medical studies and raised legitimate concerns regarding graduation, fears around self-isolating and uncertainty about returning to normal.

In these challenging times, improving student mental well-being has become a greater priority in order to reduce the known consequences of ongoing psychological distress and minimize any negative effects of the pandemic on mental health. In the medical student literature, effective factors in alleviating stress include resilience training^6^; having social support and emotional resilience^5^; proactively participating in self-care activities such as exercise, good diet and engaging in fulfilling interpersonal relationships^7^; and having personal character traits such as joy, self-efficacy and optimism.^8^ The presence of these factors can buffer against stressful and emotional situations, especially students who are prone to mental health problems.

From the study results, we know that, despite these adversities, our students have been proactive in many domains and are motivated to engage in a wide range of activities to stay busy and socially connected through difficult times. However, while many students have the capacity to adopt protective strategies and behaviours, some remain vulnerable to ongoing stress caused by the pandemic. The Medical School, in conjunction with the Western Australia Medical Students’ Society, has sought to allay students’ concerns by providing regular communications and updates regarding changes to teaching delivery (lectures, tutorials, placements), assessments and the resumption of clinical placements.

Limitations of the study include its cross-sectional design and relatively low response rate (37.5%), meaning that the full range of impacts and concerns relating to the pandemic may not have been captured. This is a longitudinal study and students will be re-surveyed over the coming months to gain a long-term perspective of the COVID-19 impact.

In conclusion, this study has provided insights of the impact of COVID-19 on our students’ well-being and provided an opportunity to address their concerns. We hope that results help other medical schools by raising awareness of how the pandemic impacts on well-being, thus enabling the provision of appropriate support. Ongoing research to monitor student well-being over time as the effects of the pandemic endure, will be important to minimize its negative impacts.

## References

[1] beyondblue. National mental health survey of doctors and medical students, https://www.beyondblue.org.au/docs/default-source/research-project-files/bl1132-report—nmhdmss-full-report_web (2013, accessed 29 May 2020).

[2] Rotenstein L Ramos M Torre M, et al Prevalence of depression, depressive symptoms, and suicidal ideation among medical students: a systematic review and meta-analysis. JAMA 2016; 316: 2214–2236. 27923088 10.1001/jama.2016.17324 PMC5613659

[3] Weber J Skodda S Muth T, et al Stressors and resources related to academic studies and improvements suggested by medical students: a qualitative study. BMC Med Educ 2019; 19: 312. 31429744 10.1186/s12909-019-1747-z PMC6701044

[4] Drybye L Shanafelt T. A narrative review on burnout experienced by medical students and residents. Med Educ 2016; 50: 132–149. 26695473 10.1111/medu.12927

[5] Bore M Kelly B Nair B. Potential predictors of psychological distress and well-being in medical students: a cross-sectional pilot study. Adv Med Educ Pract 2016; 7: 125–135. 27042156 10.2147/AMEP.S96802 PMC4780718

[6] Bacchi S Licinio J. Resilience and psychological distress in psychology and medical students. Acad Psychiatry 2017; 41: 185–188. 27060093 10.1007/s40596-016-0488-0

[7] Ayala E Winseman J Johnsen R, et al U.S. medical students who engage in selfcare report less stress and higher quality of life. BMC Med Educ 2018; 18: 189. 30081886 10.1186/s12909-018-1296-x PMC6080382

[8] Heinen I Bullinger M Kocalevent R. Perceived stress in first year medical students - associations with personal resources and emotional distress. BMC Med Educ 2017; 17: 4. 28056972 10.1186/s12909-016-0841-8 PMC5216588

